# Dysregulation of miR-192-5p in acute pancreatitis patients with nonalcoholic fatty liver and its functional role in acute pancreatitis progression

**DOI:** 10.1042/BSR20194345

**Published:** 2020-05-28

**Authors:** Yang Hu, Yongming Yu

**Affiliations:** 1 Department of Gastroenterology, The First Affiliated Hospital of Nanchang University, Nanchang, Jiangxi 330006, China; 2 Department of Gastroenterology,Jiangxi Province Hospital of Integrated Chinese and Western Medicine, Nanchang, Jiangxi 330003, China

**Keywords:** acute pancreatitis, diagnosis, inflammation, microRNA-192-5p, nonalcoholic fatty liver disease, proliferation

## Abstract

**Background:** Nonalcoholic fatty liver disease (NAFLD) is a frequent metabolic disease and has been demonstrated to contribute to the severity of acute pancreatitis (AP). The present study aimed to investigate the aberrant expression of microRNA-192-5p (miR-192-5p) in AP patients with NAFLD, and further analyze the clinical significance and biological function of miR-192-5p in AP progression.

**Methods:** Expression of miR-192-5p was estimated using quantitative real-time PCR (qRT-PCR). Diagnostic value of miR-192-5p was evaluated by the receiver operating characteristic curve (ROC). The effects of miR-192-5p on cell proliferation, apoptosis and inflammatory response of pancreatic acinar cells were further assessed by CCK-8 assay, flow cytometry and enzyme-linked immunosorbent assay (ELISA).

**Results:** Circulating miR-192-5p was decreased in AP patients with NAFLD compared with those patients without NAFLD and healthy controls (*P*<0.05). The down-regulated expression of miR-192-5p had a relative high diagnostic accuracy to distinguish the AP patients with NAFLD from the cases without NAFLD. Furthermore, the overexpression of miR-192-5p in pancreatic acinar cells led to the decreased cell proliferation, increased cell apoptosis and suppressed inflammatory reaction (all *P*<0.05).

**Conclusion:** Collectively, all data indicated that serum expression of miR-192-5p in AP patients with NAFLD is significantly decreased and serves as a candidate diagnostic biomarker. The up-regulation of miR-192-5p in pancreatic acinar cell leads to increased cell apoptosis and decreased inflammatory response, suggesting the potential of miR-192-5p as a therapeutic target of AP.

## Background

Acute pancreatitis (AP) is an inflammatory disease occurring in pancreas, which can progress to multiple organ failure with increasing morbidity and high mortality rate [1]. Abdominal pain is the major clinical manifestation of AP [2]. It is reported that pancreatic enzymes are abnormally activated in AP, leading to the autodigestion of pancreas [3]. Most of AP is determined as mild AP (MAP), accounting for approximately 80% of all AP cases with self-limitation and good prognosis. However, approximately 20% patients suffering from severe AP (SAP), which is considered a clinical health threat with high mortality [4]. Therefore, early diagnosis is of great importance in patients with AP. Emerging studies provide evidence that pancreatitis leads to severe pathological changes in pancreatic acinar cells, and the damage of cells promotes the release of enzymes and organelles, which resulted in the occurrence of SAP [5]. Thus, strategies to promote pancreatic acinar cell apoptosis may have therapeutic potential in the treatment of AP.

Nonalcoholic fatty liver disease (NAFLD) is a type of metabolic disease characterized by excessive accumulation of hepatic fat [6]. It is one of the major risk factors of AP and contributes to the severity of AP [7]. Previous evidence has reported that NAFLD could be involved in the development of AP by obesity, Kuppfer cell, oxidative stress and hyperlipermia [8]. However, the understanding about the precise molecular mechanisms underlying the promoting effects of NAFLD on AP development remains limited. MicroRNAs (miRNAs) are a group of small noncoding RNAs with important regulatory effects on gene expression at post-transcriptional levels [9]. Some aberrantly expressed miRNAs have been found to be related with the occurrence and development of AP and NAFLD, providing candidate diagnostic biomarkers or therapeutic targets [10,11]. Among the functional miRNAs in NAFLD progression, several members have been found to be also involved in the development of AP, such as miR-21 [12,13] and miR-27a [14,15]. A study by Liu et al. reported that microRNA-192-5p (miR-192-5p) was deregulated in NAFLD and involved in the regulation of lipid synthesis [16]. Another study highlighted the regulatory effect of miR-192-5p on inflammatory response and progression of NAFLD [17]. However, the relationship between miR-192-5p and AP is rarely reported.

In the present study, we sought to examine the serum expression of miR-192-5p in AP patients with or without NAFLD and evaluate its association with AP severity and diagnosis. In addition, a pancreatic acinar cell line was used to analyze the effects of miR-192-5p on cell proliferation, apoptosis and inflammatory response to further uncover the biological function in AP progression.

## Materials and methods

### Patients and serum collection

The present study recruited 60 healthy volunteers, 60 NAFLD patients and 120 AP patients from the First Affiliated Hospital of Nanchang University from 2015 to 2017. The basic information of the study population was recorded in Table 1. The participants in the three groups were age- and gender-matched, and there was no significant difference for body mass index (BMI) distribution among three groups. Among the AP patients, there were 60 cases with NAFLD (AP/NAFLD+) and 60 cases without NAFLD (AP/NAFLD−). The healthy volunteers were enrolled from the individuals who received physical examination in the First Affiliated Hospital of Nanchang University, who exhibited no evidence of disease. The patients with NAFLD were diagnosed following the criteria of liver steatosis in at least 5% of hepatocytes. The diagnosis of AP was performed based on two of the following three clinical characteristics: (i) abdominal pain consistent with AP; (ii) the levels of amylase and/or lipase were more than three times of the upper limit of normal; (iii) observation of AP characteristics by an abdominal computed tomography (CT) examination. To evaluate the severity of AP, the Acute Physiology and Chronic Health Evaluation II (APACHE II) score and Ranson score of the AP patients were recorded. The 120 AP patients were divided into MAP and SAP by the APACHE II score, Ranson score, CT examination data, status of organ failure and local complication. Venous blood was collected from the study population and serum was isolated by centrifugation. None of the patients received any therapy before sampling. The experimental procedures were approved by the Ethics Committee of the First Affiliated Hospital of Nanchang University and each participant provided written informed consent.

**Table 1:** Basic information of the study population

Features	Health (*n*=60)	NAFLD (*n*=60)	AP (*n*=120)	*P*-value
Age (years)	52.30 ± 10.65	52.68 ± 11.37	51.83 ± 11.78	0.890
Gender (male/female)	33/27	36/24	69/51	0.858
BMI (kg/m^2^)	24.96 ± 3.30	25.23 ± 3.66	25.54 ± 3.53	0.561

### Cell culture and AP cell model construction

A rat pancreatic acinar cell line AR42J was purchased from the American Type Culture Collection (ATCC) and cultured in Dulbecco’s modified Eagle’s medium (DMEM; Gibco, Grand Island, NY) supplemented with 10% fetal bovine serum (FBS; Gibco) at 37°C in a humidified atmosphere of 5% CO_2_. The AR42J cells were seeded in six-well plates with a density of 1 × 10^6^ cells/well, and 10 nM cerulein (Sigma, Louis, MO) was added into the cells to construct an AP cell model. After incubation for 6 h, cells were collected for further experiments.

### Cell transfection

To achieve *in vitro* regulation of miR-192-5p, cell transfection was performed with miR-192-5p mimic or mimic negative control (NC; GenePharma, Shanghai, China) by Lipofectamine 3000 (Invitrogen, Carlsbad, CA, U.S.A.) following the manufacturer’s protocols. The subsequent cell biological function examination was detected at 48 h after the transfection.

### RNA extraction and quantitative real-time PCR

Total RNA was isolated from the serum and cells using TRIzol reagent (Invitrogen, Carlsbad, CA, U.S.A.). Single-stranded cDNA was synthesized from RNA by a reverse transcription reagent kit (Invitrogen, Carlsbad, CA, U.S.A.) according to the manufacturer’s instructions. The expression of miR-192-5p was examined using quantitative real-time PCR (qRT-PCR), which was performed using a SYBR Green PCR kit (TaKaRa, Dalian, China) on a 7500 Real-Time PCR System (Applied Biosystems, U.S.A.). U6 was used as the endogenous control for miR-192-5p. The final relative expression value was calculated using the 2^−ΔΔ*C*_t_^ method.

### CCK-8 assay

Cell proliferation was analyzed using a CCK-8 kit (Dojindo Laboratories, Kumamoto, Japan). After cell transfection and cerulean treatment, AR42J cells were seeded into 96-well plates with a cell density of 2 × 10^3^ cells/well and cultured at 37°C for 72 h. The CCK-8 reagent was added into the cells at a same time point everyday, followed by a further 2-h incubation. The absorbance at 450 nm was measured by a microplate reader (Omega BioTek, GA, U.S.A.) to reflect cell proliferation ability.

### Flow cytometry assay

Cell apoptosis was assessed using an FITC Annexin V Apoptosis Detection kit (BD Biosciences, Franklin Lakes, NJ). AR42J cells were collected and washed twice using PBS, then were digested by trypsin. The cells were stained by binding buffer, then incubated with annexin V-FITC and propidium iodide (PI) in a dark environment for at 37°C for 10 min. A FACS Calibur flow cytometer (BD, Biosciences) was used to detect the final cell apoptosis rate.

### Enzyme-linked immunosorbent assay

The inflammatory responses in AP patients and cell model were analyzed by measuring the levels of proinflammatory cytokines in serum samples and cell culture supernatants. The enzyme-linked immunosorbent assay (ELISA) kits (Boster Biotechnology Company, Wuhan, China) were used to detect the levels of interleukin (IL)-1β, IL-6 and tumor necrosis factor (TNF)-α following the manufacturer’s protocols.

### Western blot analysis

Total proteins were extracted in cold by using RIPA lysis buffer (Beyotime Biotechnology, China), and the protein concentration was measured by the BCA Assay Kit (Thermo Scientific). Protein fractions were separated with sodium dodecyl sulfate/polyacrylamide gel electrophoresis gels (10%) and then transferred on to polyvinylidene difluoride (PVDF) membrane (Millipore, Bedford, MA, U.S.A.). After blocking with 5% BSA for 2 h at 4°C, membranes were incubated with specific primary antibodies overnight at 4°C. Anti-Bax, anti-caspase-3 and anti-GAPDH were purchased from Cell Signaling Technology (Boston, MA, U.S.A.). The membranes were washed thrice and then incubated with secondary antibodies for 1 h. The protein bands were visualized using an ECL Chemiluminescence kit (Millipore, Billerica, MA, U.S.A.). GAPDH was considered as the internal control.

### Statistical analysis

All the data obtained from the present study were presented as mean ± SD and analyzed using the SPSS 18.0 software (SPSS Inc., Chicago, IL) and GraphPad Prism 5.0 software (GraphPad Software, Inc., U.S.A.). Differences between groups were analyzed using Student’s *t* test or one-way ANOVA. Correlation between parameters was assessed using a Pearson correlation assay. A receiver operating characteristic curve (ROC) was plotted to evaluate the diagnostic value of miR-192-5p. A value of *P*<0.05 was considered to be statistically significant.

## Results

### AP cell model

**Figure 1:**
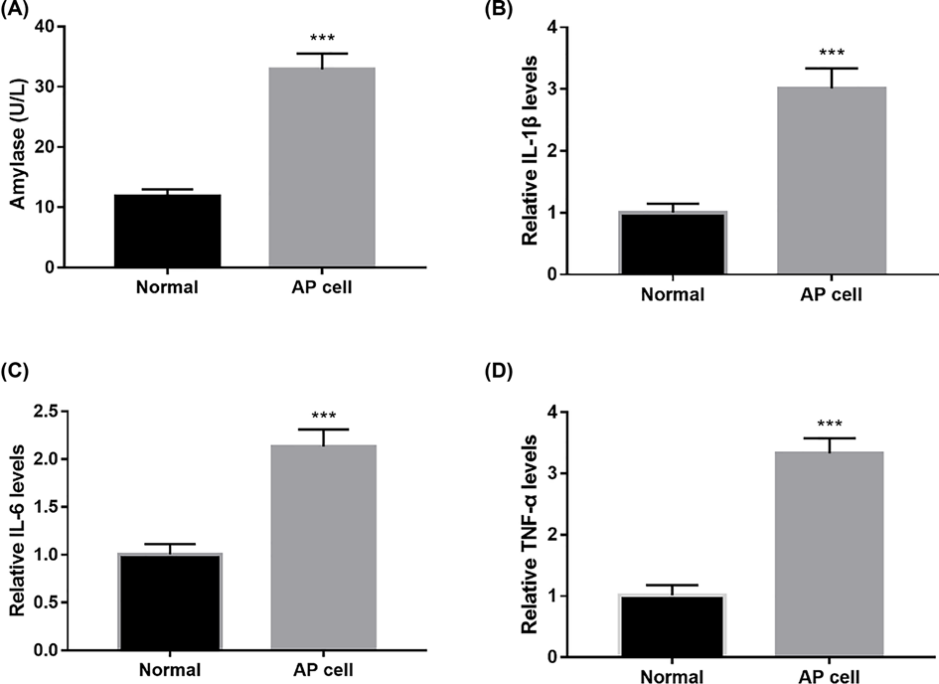
AP cell model detection After cerulein treatment, the levels of amylase (**A**), IL-1β (**B**), IL-6 (**C**) and TNF-α (**D**) increased significantly in AP model cells compared with normal cells (***P<0.001, *vs.* Normal).

As shown in Figure 1, after cerulein treatment, the levels of amylase, IL-1β, IL-6 and TNF-α increased significantly in AP model cells compared with normal cells. The results indicated that the AP cell model was established successfully.

### Expression of miR-192-5p in the patients and AP model cells

**Figure 2:**
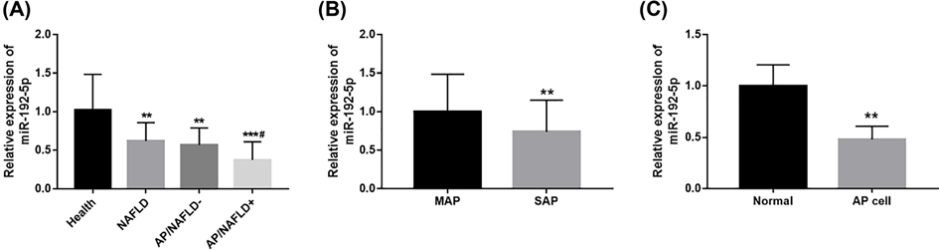
Serum expression of miR-192-5p in AP patients (**A**) The expression of miR-192-5p was decreased in NAFLD and AP patients compared with healthy controls (**P<0.01, ***P<0.001 *vs.* Healthy; ^#^*P*<0.05 *vs.* NAFLD). (**B**) miR-192-5p expression was lower in SAP group than the MAP group (**P<0.01). (**C**) miR-192-5p expression was decreased in AP model cells compared with the normal cells (**P<0.01).

Expression of miR-192-5p in serum samples collected from the participants was measured by qRT-PCR. As expected, a decreased expression of miR-192-5p was observed in patients with NAFLD compared with the healthy controls (*P*<0.01, Figure 2A). Interestingly, we found that the expression of miR-192-5p also reduced in AP patients compared with the healthy controls (*P*<0.01), and that miR-192-5p had the lowest expression in AP/NAFLD+ patients than that in the healthy control, NAFLD and AP/NAFLD patients (all *P*<0.05). In the 120 AP patients, 42 cases were diagnosed with SAP and the rest 78 cases were MAP. By the comparison between the two groups, we found that miR-192-5p was lower in SAP group than the data in MAP group (*P*<0.01, Figure 2B), implying that miR-192-5p might be involved in the development of AP. In addition, the present study used cerulean to construct an AP cell model, and found that the expression of miR-192-5p was inhibited by cerulean in AR42J cells compared with the normal cells (*P*<0.01, Figure 2C).

### Diagnostic performance of miR-192-5p

**Figure 3:**
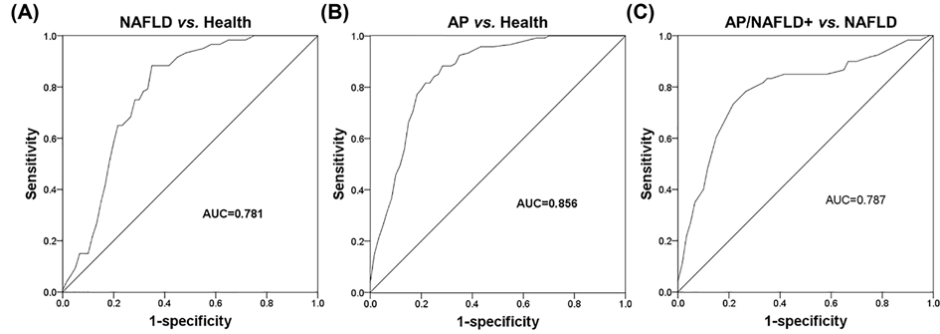
Diagnostic performance evaluation for serum miR-192-5p using the ROC analysis (**A,B**) Serum miR-192-5p expression can be used to distinguish NAFLD and AP patients from the healthy controls (AUC). (**C**) Serum miR-192-5p had a diagnostic potential to screen AP patients with NAFLD from NAFLD patients.

The present study further evaluated the clinical significance of miR-192-5p in diagnosis according to the ROC analysis. As shown in Figure 3A,B, the ROC curves revealed that serum miR-192-5p could distinguish NAFLD and AP patients from healthy individuals with a good diagnostic performance (area under the curve (AUC) of 0.781 for NAFLD patients; AUC of 0.856 for AP patients). Moreover, the ability of miR-192-5p to screen AP/NAFLD+ cases from NAFLD patients was also evaluated, and the results in Figure 3C indicated that serum miR-192-5p had a relatively high diagnostic accuracy with an AUC of 0.787.

### Association of miR-192-5p with the clinical characteristics of AP patients

To facilitate the relationship analysis, the expression of miR-192-5p was divided into low and high expression group based on its median value. The data listed in Table 2 showed that miR-192-5p was associated with patients APACHE II score (*P*=0.001) and Ranson score (*P*<0.001). No significant relationship was found between miR-192-5p and age, gender and BMI of the AP patients (all *P*>0.05).

**Table 2:** Association of miR-192-5p expression with clinical data of AP patients

Features	Total number, *n*=120	miR-192-5p expression		*P*-values
		Low (*n*=68)	High (*n*=52)	
Age (years)				0.682
<50	51	30	21	
≥50	69	38	31	
Gender				0.970
Female	51	29	22	
Male	69	39	30	
BMI (kg/m^2^)				0.064
<24	42	19	23	
≥24	78	49	29	
APACHE II score				0.001
<8	70	30	40	
≥8	48	36	12	
Ranson score				<0.001
<3	46	15	31	
≥3	74	53	21	

### Correlation of miR-192-5p with serum amylase and proinflammatory cytokine levels in AP patients

**Figure 4:**
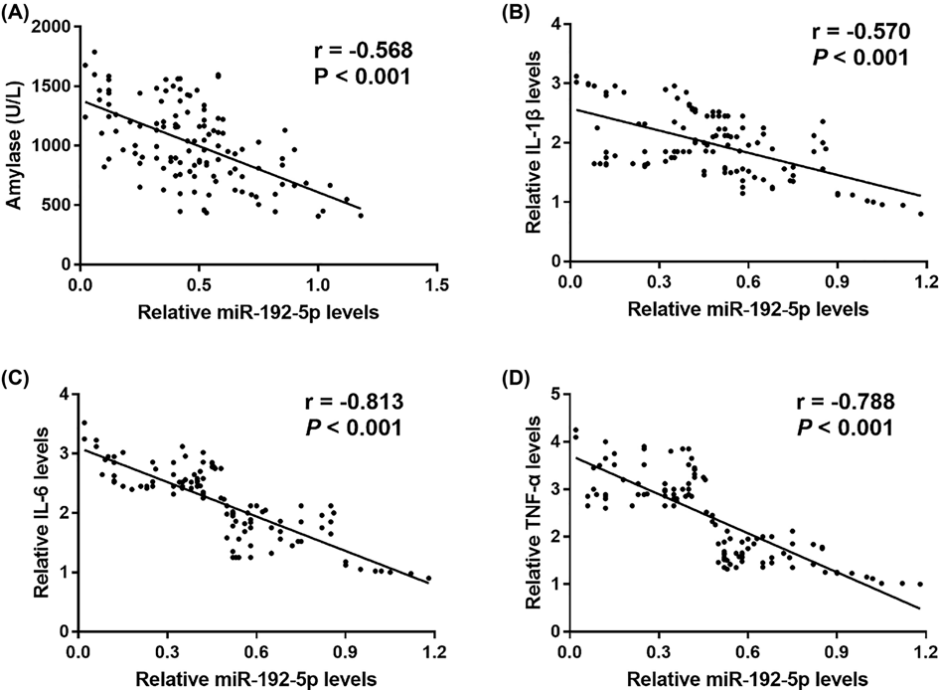
Correlation of miR-192-5p with serum amylase and serum proinflammatory cytokine levels in AP patients (**A**) Serum miR-192-5p levels were significantly negatively associated with serum amylase levels. The serum miR-192-5p expression was negatively correlated with serum IL-1β (**B**), IL-6 (**C**) and TNF-α (**D**) in patients with AP.

Considering the important role of serum amylase in the diagnosis of AP, the study investigated the correlation of serum miR-192-5p levels with serum amylase in AP patients. It was noted that serum miR-192-5p levels were significantly negatively associated with serum amylase levels (r = −0.568, *P*<0.001, Figure 4). To verify whether miR-192-5p was related with the inflammatory response of AP, the present study further analyzed the correlation of miR-192-5p with proinflammatory cytokines. The data in Figure 4 showed that serum miR-192-5p levels were negatively correlated with the levels of IL-1β (r = −0.570, *P*<0.001), IL-6 (r = −0.813, *P*<0.001) and TNF-α (r = −0.788, *P*<0.001) in AP patients (both *P*<0.001).

### miR-192-5p inhibits pancreatic acinar cell proliferation and promotes cell apoptosis

**Figure 5:**
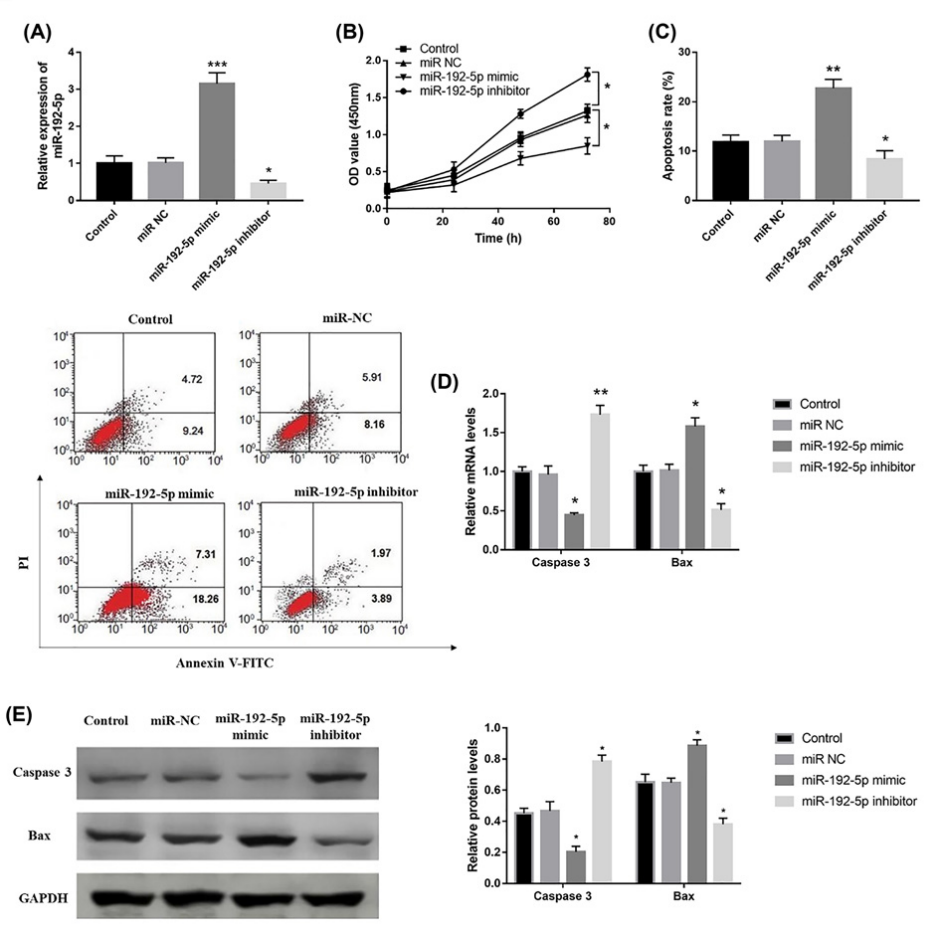
Regulatory effects of miR-192-5p on AP cell proliferation and apoptosis (**A**) The expression of miR-192-5p in AP cells was up-regulated by the miR-192-5p mimic, but was down-regulated by miR-192-5p inhibitor transfection (***P<0.001 *vs.* Control). (**B**) The overexpression of miR-192-5p inhibited AP cell proliferation, while miR-192-5p down-regulation promoted cell proliferation (*P<0.05 *vs.* Control). (**C**) The AP cell apoptosis rate was enhanced by the overexpression of miR-192-5p, but was inhibited by down-regulation of miR-192-5p (**P<0.01 *vs.* Control). (**D**) The mRNA levels of apoptosis-related proteins. The levels of Caspase 3 were down-regulated by miR-192-5p mimic transfection, which were increased by miR-192-5p inhibitor transfection. The mRNA levels of Bax were elevated by miR-192-5p mimic transfection, which were included by miR-192-5p inhibitor transfection (*P<0.05, **P<0.01, *vs.* Control). (**E**) The apoptosis-related protein levels, including Caspase 3 and Bax. Western blots were repeated three times for each protein sample (*P<0.05, *vs.* Control).

Considering the dysregulation of miR-192-5p in AP patients, its biological function in PA pathogenesis was further investigated *in vitro*. In the AP model cells, the expression of miR-192-5p was significantly elevated in the cells transfected with the miR-192-5p mimic, but was down-regulated when transfected with miR-192-5p inhibtor (*P*<0.001, Figure 5A). The cell proliferation shown in Figure 5B revealed that the overexpression of miR-192-5p in AP cells led to inhibited cell proliferation, while down-regulation of miR-192-5p significantly promoted cell proliferation (all *P*<0.05). And apoptosis data shown in Figure 5C–E revealed that the overexpression of miR-192-5p in AP cells promoted cell apoptosis, which was reflected by cell apoptosis rate and the protein levels of apoptosis related proteins, including caspase 3 and Bax (all *P*<0.05). However, down-regulation of miR-192-5p remarkably inhibited cell apoptosis (all *P*<0.05, Figure 5C–E).

### miR-192-5p suppresses inflammation in pancreatic acinar cells

**Figure 6:**
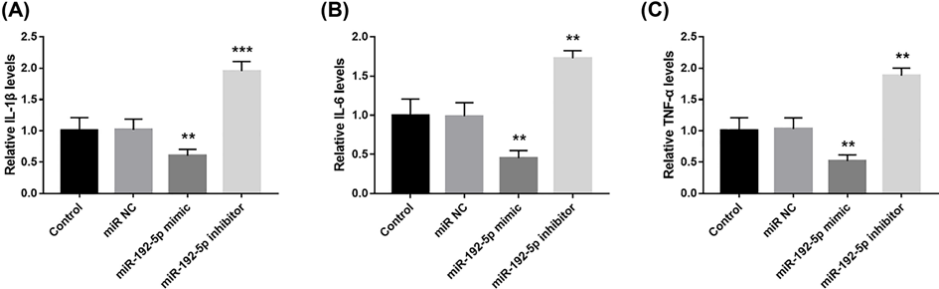
Overexpression of miR-192-5p inhibited the inflammatory response in AP model cells The levels of IL-1β (**A**), IL-6 (**B**) and TNF-α (**C**) in AP cell culture supernatant were reduced by the up-regulation of miR-192-5p, which were elevated by down-regulation of miR-192-5p (**P<0.01, ***P<0.001, *vs.* Control).

By examining the levels of proinflammatory cytokines in cell culture supernatants, the inflammatory responses were activated in the AP model cells, as evidenced by the elevated IL-1β, IL-6 and TNF-α levels (all *P*<0.001, Figure 6). What worth noting was that the increased IL-1β, IL-6 and TNF-α levels in AP model were significantly inhibited by the overexpression of miR-192-5p, which were aggravated by down-regulation of miR-192-5p (all *P*<0.001), indicating the regulatory effect of miR-192-5p on inflammation in AP progression.

## Discussion

NAFLD is considered to be a major risk factor for the occurrence of AP. The present study focused on the clinical value and role of miR-192-5p in AP. Numerous studies have highlighted the important roles of aberrant miRNAs in various human diseases [18]. The deregulated expression levels of miRNAs in serum collected from patients have been considered to be the promising diagnostic tools, and the functional miRNAs have potentials to serve as therapeutic target due to their regulatory effects on disease progression [19,20]. In AP patients, the elevated expression of miR-551b-5p has been reported to related with the inflammation and disease progression [21], the up-regulation of circulating miR-29a has been found to be associated with disease severity and predict poor prognosis [22], and the increased levels of miR-7, miR-9, miR-122 and miR-141 have been identified as a group of noninvasive biomarkers [23]. In addition, the investigations on the role of miR-21-3p and miR-148a gave evidence that the functional miRNAs were involved in the pathogenesis of AP and might provide novel insight in the targeted therapy [13,24]. The aforementioned research data implied that identification of novel miRNAs that abnormally expressed in disease progression could improve the diagnosis and treatment of AP.

As a major risk factor of AP, NAFLD can contribute to the development and severity of AP through obesity, Kuppfer cell, oxidative stress and hyperlipermia [8]. The inflammatory responses have been found to be significantly enhanced by NAFLD in AP patients [25]. However, the understanding about the molecular mechanisms for the promoting effect of NAFLD on AP progression remains limited. Some miRNAs with aberrant expression have been found to participate the development and progression of NAFLD. For example, the increased expression of miR-181b in NAFLD acted as a regulator of the steatosis by targeting SIRT1 in the progression of NAFLD [26]. The aberrant miR-26a could contribute to the fatty acid and sterol metabolism of NAFLD in the cell model that was constructed by free fatty acid in HepG2 cells [27]. Among the identified functional miRNAs in NAFLD, some of them have also been found to be involved in the pathogenesis of AP. For instance, the deregulated miR-21 in NAFLD has been found to promote the pancreatic injury and inhibit necrotic acinar cell apoptosis in AP rats [12,13]. miR-27a not only regulated the hepatic lipid metabolism of NAFLD but also modulated the pancreatic acinar cell apoptosis and inflammation in AP [14,15]. miR-192-5p has been reported to regulate the lipid synthesis in NAFLD [16], but no information has been found for miR-192-5p in AP pathogenesis.

In the present study, serum expression of miR-192-5p was verified in NAFLD patients and further checked in the patients with AP. The data revealed that serum miR-192-5p expression was decreased in NAFLD and AP patients compared with healthy controls, and AP patients with positive NAFLD had a lower miR-192-5p expression than the simple NAFLD patients. The subsequent clinical significance evaluation results indicated that serum miR-192-5p served as a candidate diagnostic biomarker for NAFLD and AP patients, and that miR-192-5p had a considerable diagnostic potential to distinguish AP patients with NAFLD from the simple NAFLD patients. Thus, we deduced that miR-192-5p might be involved in the promoting effect of NAFLD on the progression of AP, and has predictive value for the occurrence of AP in patients with NAFLD. Furthermore, the relationship between miR-192-5p and AP severity was examined, and serum miR-192-5p expression was found to be increased in SAP patients compared with MAP patients, and was associated with AP severity indicators (APACHE II score and Ranson score). Additionally, a negative correlation of miR-192-5p with AP patients’ inflammation was found, and the overexpression of miR-192-5p in AP model cells led to a significant inhibition in the inflammatory response. Collectively, miR-192-5p might be involved in the pathogenesis of AP and serve as a potential diagnostic biomarker. These results provide a certain theoretical basis for the early diagnosis and treatment of AP in future.

It is well known that the occurrence of pancreatitis contributes to the severe pathological changes in pancreatic acinar cells, leading to the development of AP [28]. The damage in pancreatic acinar cells accelerates cell necrosis and leads to the release of enzymes and organelles to aggravate the inflammation of AP [15]. Thus, the cell apoptosis of pancreatic acinar cells is considered a self-protection strategy. The regulatory effects of miR-192-5p on cell apoptosis have been reported in myocardial ischemia/reperfusion injury and osteosarcoma [15,29]. In the current study, we found that the up-regulation of miR-192-5p in pancreatic acinar cells could promote the cell apoptosis rate in AP model cells, leading us to draw a conclusion that miR-192-5p might be involved in the pathogenesis of AP by regulating pancreatic acinar cell apoptosis. It is well known that the dysregulation of miRNA always functions through regulating their target genes expression. In a study about asthma, miR-192-5p is reported to attenuate autophagy in asthma by targeting autophagy-related 7 (ATG7), overexpression of miR-192-5p is suggested to down-regulate ATG7 levels [30]. ATG7 plays an important role in autophagy, and lack of ATG7 suppresses the autophagic processes [31]. A study by Zhou et al. has reported that deletion of ATG7 in pancreatic acinar cells was sufficient to induce strong acinar cell death associated with AP [32]. The present results indicated that overexpression of miR-192-5p promoted pancreatic acinar cell apoptosis. Combined with the previous evidence, we concluded that miR-192-5p might regulating pancreatic acinar cell apoptosis in AP progression, and ATG7 might be involved in the regulatory role. However, further studies are needed to verify our hypothesis.

In conclusion, all the data obtained from the present study indicated that serum expression of miR-192-5p in AP patients with positive NAFLD is significantly decreased and can be used as a candidate diagnostic biomarker and an indicator to predict AP in NAFLD patients. The increased miR-192-5p is associated with the severity of AP and may be involved in the disease pathogenesis by regulating inflammatory response and pancreatic acinar cell apoptosis, indicating the potential of miR-192-5p as a novel therapeutic target in AP treatment. Although the present study provides a novel insight into the diagnosis and treatment of AP by regarding miR-192-5p, the molecular mechanisms involving in the regulatory effects of miR-192-5p in AP progression remain unclear, which is a limitation of the present study and warrant further investigations.

## Data Availability

The datasets used and/or analyzed during the current study are available from the corresponding author on reasonable request.

## Abbreviations

AP: acute pancreatitis
APACHE II: Acute Physiology and Chronic Health Evaluation II
ATG7: autophagy-related 7
AUC: area under the curve
ATCC: American type culture collection
ANOVA: analysis of variance
ATG7: autophagy related 7
BMI: body mass index
Bax: BCL2-Associated X protein
CT: computed tomography
CCK-8: Cell Counting Kit-8
DMEM: Dulbecco’s modified Eagle’s medium
ELISA: enzyme-linked immunosorbent assay
FBS: fetal bovine serum
GAPDH: glyceraldehyde-3-phosphate dehydrogenase
IL: interleukin
MAP: mild AP
miRNA: microRNA
miR-192-5p: microRNA-192-5p
NC: negative control
NAFLD: nonalcoholic fatty liver disease
PI: propidium iodide
PVDF: polyvinylidene difluoride
qRT-PCR: quantitative real-time PCR
ROC: receiver operating characteristic curve
SAP: severe AP
SD: standard deviation
SIRT1: silent Information regulator 1
TNF: tumor necrosis factor
